# Identifying lncRNA–Protein Interactions in Hematopoietic Progenitor Cells by Hybridization Capture and Mass Spectrometry

**DOI:** 10.1021/acs.jproteome.5c00334

**Published:** 2025-08-01

**Authors:** Yuling Dai, Jeong-Ah Kim, Isabella T. Whitworth, Mark Scalf, Mabel M. Jung, Brian L. Frey, Emery H. Bresnick, Lloyd M. Smith

**Affiliations:** † Department of Chemistry, 5228 University of Wisconsin-Madison, Madison, Wisconsin 53706, United States; ‡ Wisconsin Blood Cancer Research Institute, Carbone Cancer Center, Department of Cell and Regenerative Biology, University of Wisconsin School of Medicine and Public Health, Madison, Wisconsin 53705, United States

**Keywords:** lncRNA, lncRNA–protein interaction, hematopoiesis, mass spectrometry, proteomics, hybridization capture

## Abstract

Long noncoding RNAs (lncRNAs) exert regulatory functions in a wide spectrum of biological contexts, and certain regulatory functions involve the formation of RNA–protein complexes. Discovering the structure and function of these complexes may unveil important functional insights. The *DDX41* gene encoding the DEAD-box RNA helicase 41 protein (DDX41) is subject to extensive germline genetic variation, and certain variants create a predisposition to develop myelodysplastic syndrome and acute myeloid leukemia. While the importance of *DDX41* for the control of hematopoiesis is established, many questions remain regarding the mechanisms of how *DDX41* functions in hematopoietic stem and progenitor cells. Previously, we identified a DDX41-regulated lncRNA, growth-arrest-specific 5 (*Gas5*). As the *Gas5* function in hematopoiesis is unknown, we analyzed the protein interactors of *Gas5* lncRNA using HyPR-MS (hybridization purification of RNA–protein complexes, followed by mass spectrometry). A total of 303 proteins were identified as *Gas5* lncRNA interactors, five of which were experimentally validated as *Gas5* lncRNA interactors by RNA immunoprecipitation qPCR (RIP-qPCR) analysis. The identification of protein interactors with a DDX41-regulated lncRNA establishes a foundation on which to guide future mechanistic and biological studies.

## Graphical abstract

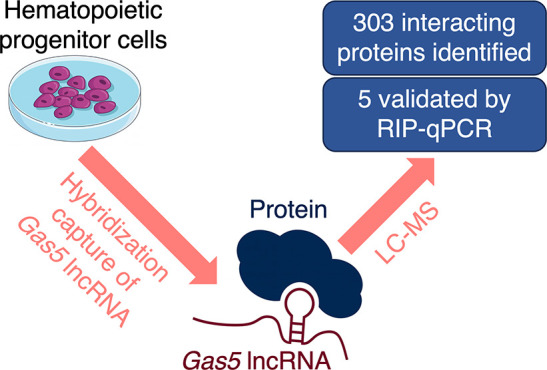

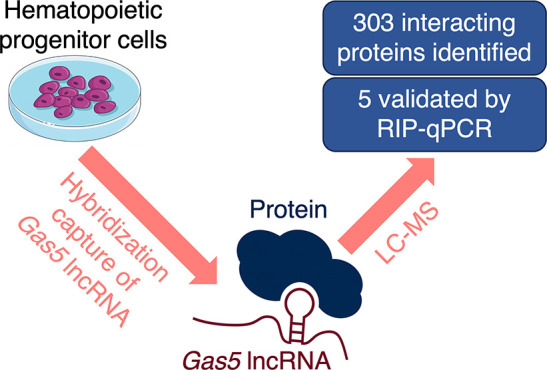

## Introduction

Two large-scale genomic projects, the Encyclopedia of DNA Elements (ENCODE)^1^ and the Functional Annotation of the Mammalian Genome (FANTOM),^2^ revealed that ∼80% of the human genome undergoes transcription to produce RNA molecules. However, a very small fraction (∼2%) of the human genome is dedicated to encoding proteins. The remaining RNA transcripts are termed noncoding RNAs, with those longer than 200 nucleotides (nt) denoted as long noncoding RNAs (lncRNAs). A previous study reported the identification of over 60,000 lncRNAs in human.^3,4^ Many lncRNAs are widely expressed and function as regulators of gene expression at multiple levels (epigenetic, post-transcriptional, and translational) in physiological and pathological processes.^4–7^

Since lncRNA mechanisms may involve protein targets, identification of specific RNA-binding proteins (RBPs) is vital for understanding the lncRNA contribution to the protein-dependent mechanism.^8,9^ RBPs, which bind RNA through one or more RNA-binding domains, determine the functional consequences of RNA binding.^10^ For example, lncRNA binding to an RBP can influence RBP post-translational modifications, such as ubiquitination and phosphorylation, to facilitate or inhibit these processes.^11^ In addition, lncRNAs can alter RBP subcellular localization and transport, including inhibiting the transport of RBPs from the nucleolus to the nucleoplasm, promoting RBP shuttling in and out of the nucleus, or recruiting RBPs to target gene promoters. Moreover, lncRNAs may promote or hinder RBP interactions with specific mRNAs, proteins, or DNA sites.^11^ Interactions between lncRNAs and RBPs have been implicated in pathogenesis, including cancer.^12,13^ Thus, identifying the proteins bound to a lncRNA can be vital for deciphering physiological and pathological mechanisms.

Biological processes reported to be lncRNA-regulated include the control of cell fate in the hematopoietic system.^14,15^ For example, lncRNAs *Xist* and *H19* play roles in maintaining hematopoietic stem cell quiescence and self-renewal. *H19* modulates these processes via an Igf2–Igfr1-dependent mechanism, and deficiency of *Xist* leads to aberrant maturation and aging-dependent loss.^16,17^ Some lncRNAs (e.g., lincRNA-EPS) are implicated in red blood cell development, while others (e.g., *EGO*) regulate eosinophil differentiation.^18,19^ *LincRNA-Cox2*, *NKILA*, *PACER*, and *Lethe* function in the innate immune response.^19–22^ Several lncRNAs are implicated in hematopoietic diseases acting as either oncogenic or tumor-suppressive factors. *BGL3* functions as a competitive endogenous RNA for the tumor suppressor gene phosphatase and tensin homologue (*PTEN*), while *H19* facilitates Bcr-Abl-mediated leukemogenesis.^23,24^ lncRNAs *IRAIN* and *RUNXOR* are implicated in acute myeloid leukemia cell growth and leukemogenesis, respectively.^25,26^ LncRNA *LUNAR1* acts as an oncogene in T-cell acute lymphoblastic leukemia, and *DLEU1/2* regulates the NF-κB signaling pathway, influencing leukemia cell survival.^27,28^ Given the importance of lncRNAs in hematopoiesis and the critical role of protein interactions in lncRNA mechanisms, identifying lncRNA–protein complexes has important implications for elucidating hematopoietic processes.

DDX41 is a DEAD/H-box helicase protein that is a highly conserved member of the RNA helicase family of metazoan proteins.^29^ DDX41 functions in post-transcriptional and translational processes, including the regulation of pre-mRNA splicing and rRNA transcription.^29,30^ DDX41 is found in the cytoplasm and nucleus^31,32^ and regulates pre-mRNA splicing, small nucleolar RNA processing, pre-rRNA processing, and interacts with R-loops.^30,32,33^ DDX41 deficiency leads to R-loop accumulation and subsequent increased dsDNA breaks, which can trigger a cGAS-STING-mediated type I IFN response.^34^ While some studies have revealed important DDX41-dependent mechanisms, e.g., biallelic *Ddx41* mutant bone marrow cells exhibit impaired small nucleolar RNA processing and ribosome function, leading to cell death of proliferating hematopoietic cells, many questions remain unanswered.^33^ Also, since DDX41 regulates RNA splicing through incompletely defined mechanisms, and germline heterozygous *DDX41* mutations create a predisposition for acute myeloid leukemia and myelodysplastic syndromes,^30,35,36^ it is instructive to consider whether lncRNAs operate in DDX41-associated hematopoietic malignancy.

To identify DDX41-regulated lncRNAs in hematopoietic progenitor cells, we conducted differential expression analysis of RNA-seq data sets obtained from three cell lines. These cell lines were HoxB8-immortalized wild-type murine fetal liver hematopoietic progenitors (WT), *Ddx41* heterozygous mutant progenitors (HET), and these HET cells subjected to genetic rescue in which DDX41 was expressed with a retrovirus also expressing green fluorescent protein (GFP) to achieve the same DDX41 level as WT cells.^37^ Our analysis identified one DDX41-regulated lncRNA, *Gas5*, which we reported previously to be DDX41-regulated.^37^ The *Gas5* lncRNA was first identified in a cDNA library enriched with RNA sequences expressed predominantly in growth-arrested cells.^38^ Subsequent studies provided evidence that *Gas5* is crucial for normal growth arrest, decelerates the cell cycle, and regulates apoptosis.^39–41^ *Gas5* has been observed to be downregulated in various malignancies including non-small-cell lung cancer, breast cancer, and pancreatic cancer.^39,42–44^ In addition, *Gas5* has been suggested to modulate biological functions by mediating a couple of key cellular signaling pathways, including the p53 network, the mammalian target of rapamycin (mTOR) pathway, and the glucocorticoid response element.^41,43–47^ Although *Gas5* has been studied in different cancer cell lines, there are many unanswered questions as to how it functions in the hematopoietic system, and answers to these questions may be revealed by the proteins interacting with *Gas5*.

Mass spectrometry (MS) has been well developed and widely utilized for protein identification and quantification, demonstrating excellent performance across various fields.^48–52^ In the current study, we used a hybridization capture strategy (HyPR-MS, hybridization purification of RNA–protein complexes, followed by MS) that enables the specific capture of cross-linked RNA–protein complexes and identification of the associated proteins.^53–55^ We purified *Gas5*–protein complexes using a sequence-specific pull-down and then employed MS to identify the protein interactors. These proteins were evaluated by comparisons with proteins suggested to bind *Gas5* lncRNA in other systems, comparisons with predicted *Gas5* lncRNA protein interactors, and pathway and enrichment analysis. For validation of the proteins associated with *Gas5* lncRNA, we utilized RNA immunoprecipitation-quantitative polymerase chain reaction (RIP-qPCR), a method complementary to HyPR-MS, to isolate target proteins and analyze the associated RNAs.

## Experimental Section

### Preparation of Wild-Type, Heterozygous, and Rescued Cells

We used a previously developed assay aimed at mechanistically dissecting and clinically curating human *DDX41* patient variants.^56^ Through this assay, we dissected the functional differences between wild-type and human DDX41 variants, including human patient variants we identified in the study (R293H and K331del), previously reported recurrent pathogenic variants (G173R and R525H), and a variant of uncertain significance (VUS; G610S). We found that these mutants differ from the wild type in their activities to regulate the levels of cell surface proteins and specific RNAs, including *Gas5*, and suggested that these activities could be used for clinical curation. In this study, we analyzed the transcript isoforms of *Gas5* in WT, Ddx41 heterozygous mutant progenitors (HET), and HET-DDX41 cells subjected to genetic rescue, to explore the functional mechanism of the DDX41–*Gas5* axis on DDX41 wild-type myeloid progenitor cells. The assay utilizes Hoxb8-immortalized (hi) mouse fetal liver hematopoietic progenitor cells, which exhibit a normal myeloid progenitor cell phenotype.^57^ hi-Hematopoietic progenitor cells were generated by retroviral infection of β-estradiol-regulated HoxB8 into primary Lin^–^ cells isolated from the WT mouse fetal liver. Cells were cultured in OPTI-MEM supplemented with 10% FBS, 1% penicillin–streptomycin, 1% SCF-conditioned medium, 28.6 μM β-mercaptoethanol, 1 μM β-estradiol, and 400 μg/mL G418 (immortalized cell culture media) in a humidified 5% CO_2_ incubator at 37 °C. Since these cells are immortalized, the passage number was not counted. These cells were engineered with CRISPR/Cas9 to generate *Ddx41* ^+/−^ clonal lines with reduced endogenous DDX41 protein levels.^56^ As *Ddx41*-nullizygous mice are lethal, we used the cultured cell system in which DDX41 protein is ∼50% lower than that of hi-*Ddx41* ^*+/+*^ cells to minimize the potential for deleterious effects on cellular functions and provide a model for studying human DDX41 variants. The *Ddx41* ^+/−^ cells, which were validated by Sanger sequencing of genomic DNA surrounding the target sequence, were described previously.^56^ Semiquantitative Western blotting revealed reduced DDX41 levels in hi-*Ddx41* ^+/−^ relative to hi-*Ddx41* ^*+/+*^ cells. hi-Ddx41^+/+^ and hi-*Ddx41* ^+/−^ cells were infected with a retrovirus expressing GFP alone (empty) or GFP with DDX41 or myeloid malignancy-associated *DDX41* variants. GFP-positive cells were sorted by flow cytometry to obtain the infected cells. RNA and protein from sorted GFP-positive cells were subjected to RNA-seq, reverse transcription qPCR (RT-qPCR), and semiquantitative Western blotting.^56^ Table 1 shows the fold reduction in DDX41 levels, as measured by quantitative Western blot for the three types of cells used in the current study: wild type (*Ddx41* ^*+/+*^; WT-empty or WT), heterozygous (*Ddx41* ^+/−^; HET-empty or HET), and genetic-rescued cells (HET-DDX41).

**Table 1:** Three Types of Hematopoietic Progenitor Cells Analyzed by RNA Sequencing

cell type	genotype	vector	DDX41 level
WT (WT-empty)	*Ddx41* ^*+/+*^	empty	normal DDX41 expression
HET (HET-empty)	*Ddx41* ^+/−^	empty	∼50% lower DDX41 expression compared to WT cells
HET-WT	*Ddx41* ^+/−^	WT DDX41	similar DDX41 expression compared to WT cells

### Cross-Linking, Cell Lysis, Hybridization, Capture, and Release

The cross-linking, lysis, hybridization, capture, and release protocols are adapted from the HyPR-MS protocol of Henke et al., summarized here to include any changes.^39^ (See Supporting Information Note S1 for a list of reagents, materials, and equipment used in this work.) For HyPR-MS experiments, WT hi-hematopoietic progenitor cells, not infected with retrovirus expressing GFP, were cultured in immortalized cell culture media. These cells (small-scale: 1 × 10^6^ per condition; large-scale: 1 × 10^8^ per bioreplicate) were washed twice with phosphate-buffered saline (PBS) and then incubated in 1% formaldehyde in PBS for 10 min at room temperature (RT). Formaldehyde was quenched by incubation with 250 mM Tris–HCl buffer (pH 8.0) for 10 min at RT. Cells were rinsed with ice-cold PBS twice, pelleted by centrifugation at 1200 rpm for 5 min, flash-frozen in liquid nitrogen, and stored at −80 °C. Cells were lysed and sonicated as described. Biotinylated capture oligonucleotides (COs) targeting *Gas5* lncRNA were added to the cell lysates with negative control scrambled sequence oligonucleotides (SOs) (Supporting Information Figure S1 and Table S1). Both COs and SOs were used at 500 pmol per sample. The scrambled oligonucleotide was designed to have the same number of nucleotides and a comparable melting temperature (*T*_m_) as the CO but lacking significant complementarity to the target transcript or any other transcript in the cells. The samples were incubated at 37 °C for 3 h with gentle nutation. SeraMag streptavidin-coated magnetic speed beads (Fisher Scientific) were washed twice at RT and added to the lysate to capture the biotinylated oligonucleotide–RNA complexes. Based on a ratio of 2.2 μL beads per pmol CO, 1100 μL beads were added to each sample. The samples were rocked for 1 h before bead collection and lysate removal. The beads were washed and resuspended in a release buffer (375 mM LiCl, 50 mM Tris (pH 7.5), 0.1% LiDS, and 0.1% Triton X-100). The release buffer volume was calculated as three times the bead volume, resulting in a total of 3300 μL of release buffer per sample. Release oligonucleotides (ROs) (Supporting Information Table S1) were then added; the beads were gently rocked at RT for 30 min to release RNA–protein complexes; and the supernatant was isolated and stored at 4 °C. The release process was accomplished with *Gas5* lncRNA target RO, followed by scrambled RO. The supernatants from each release were transferred separately to new tubes, and each was divided into aliquots for RT-qPCR and mass spectrometric protein analysis.

### RNA Extraction, Reverse Transcription, and qPCR Analysis

A small fraction (2%) of each released sample was incubated overnight at 37 °C with 1 mg/mL Proteinase K (Sigma), 4 mM CaCl_2_, and 0.2% LiDS to remove proteins. The RNA was extracted from each sample using TRI Reagent (Sigma) and precipitated in 75% ethanol with 2 μL of GlycoBlue coprecipitant (ThermoFisher Scientific) at −20 °C overnight (at least 10 h). RNA was pelleted by centrifuging at max speed (20,800*g*; 4 °C; 15 min), washed with 75% ethanol, centrifuged at max speed (20,800*g*) (20 °C; 15 min), and resuspended in 15 μL of nuclease-free water (Invitrogen). Purified RNA (10 μL) was used for reverse transcription (High-Capacity cDNA Reverse Transcription Kit, Applied Biosystems) per the manufacturer’s protocol. The reverse transcription product (20 μL) was diluted with 60 μL of nuclease-free water (Invitrogen) and analyzed using sequence-specific qPCR primers and probes (Integrated DNA Technologies) and Light Cycler 480 Probes Master Mix (Roche) for the relative quantitation of RNA on a CFX96 Touch real-time PCR detection system (Bio-Rad).

### Protein Purification and Digestion

Proteins from the remainder of each capture sample were purified and digested into peptides with trypsin using an adapted version of eFASP, as described.^58^ Each release sample was brought to 0.1% deoxycholic acid (DCA) and 8 M urea and passed through the filter, and the eluant was discarded. Exchange buffer (8 M urea and 0.1% deoxycholic acid (DCA)) was added to the filter, and the tube was centrifuged at 14,000*g* for 10 min. This was repeated three times. Reduction was performed by adding 200 μL of reducing buffer (8 M urea and 20 mM dithiothreitol (DTT)), followed by incubation at 37 °C for 30 min. The buffer was then removed by centrifugation at 14,000*g* for 10 min. Alkylation was carried out by adding 200 μL of alkylation buffer (8 M urea, 50 mM iodoacetamide, and 50 mM ammonium bicarbonate) and incubating at RT in the dark for 30 min. Excess alkylating reagent was removed via centrifugation, and the filters were rinsed multiple times with the exchange buffer to eliminate residual reagents. The filters were transferred to clean, passivated collection tubes, and 100 μL of digestion buffer (1 M urea, 50 mM ammonium bicarbonate, and 0.1% DCA) containing 0.3 μg of trypsin was added per sample. The tube closure was wrapped with Parafilm to prevent evaporation, and digestion was carried out overnight at 37 °C without agitation.

Following incubation, the Parafilm was removed, and the sample was centrifuged at 14,000*g* for 10 min to collect the flow-through. To enhance peptide recovery, 50 μL of 50 mM ammonium bicarbonate was added to the filter, followed by centrifugation at 14,000*g* for 10 min. This step was repeated once more. All filtrates were collected in the same tube, and the resulting filtrate was transferred to a new low-binding tube with a secure seal. To facilitate detergent removal, 200 μL of ethyl acetate containing trifluoroacetic acid (TFA) was added, ensuring thorough mixing before addition. Specifically, 21 μL of 10% TFA was used to give a concentration of 0.5%, based on a total solution volume of 400 μL. The mixture was shaken for 1 min using a vortex mixer, followed by centrifugation at 15,700*g* for 2 min. The top organic layer (ethyl acetate) was carefully removed. This extraction step was repeated twice, each time adding 200 μL of ethyl acetate without additional TFA. Finally, the peptide sample was dried to completion using a SpeedVac concentrator for approximately 2.5 h, and then the sample was reconstituted in 150 μL of 0.1% TFA.

To remove salts from the sample, an OMIX C18 solid-phase extraction pipet tip (Agilent) was conditioned with 70% ACN, 0.1% TFA, and then equilibrated with 0.1% TFA. The peptide sample was loaded onto the C18 solid phase by repeated passing of the 150 μL sample over the cartridge. The OMIX pipet tip was then rinsed with 0.1% TFA 10 times, followed by peptide elution in 150 μL of 70% ACN and 0.1% TFA. The samples were dried using a SpeedVac and reconstituted in 10 μL of 95:5 H_2_O/ACN and 0.2% formic acid and analyzed as described below.

### MS of Peptides

Eight of the 10 μL of each sample was analyzed using a LC–MS/MS system consisting of an ultrahigh-pressure liquid chromatography system coupled to an Orbitrap Fusion Lumos mass spectrometer (ThermoFisher Scientific). A 100 μm i.d. fused silica capillary nanocolumn packed with 20 cm of C18 beads (1.7 μm diameter, 130 Å pore size, Waters BEH) and an emitter tip pulled to approximately 1 μm using a laser puller (Sutter Instruments) were used for UHPLC separation of peptides. Bioreps 1 and 2 used an Easy-nLC 1200 (ThermoFisher Scientific) instrument for separation. Peptides were loaded onto the column in buffer A (H_2_O and 0.2% formic acid) at a pressure of 300 bar. Peptides eluted over 120 min at a flow rate of 350 nL/min with the following gradient, where buffer B was 80% acetonitrile, 0.2% formic acid: time 1 min, 5% buffer B; time 52 min, 30% buffer B; time 80 min, 42% buffer B; time 90 min, 55% buffer B; time 95 to 100 min, 85% buffer B; time 101 to 120 min, equilibrated at 0% buffer B. The nanocolumn was held at 60° C using a column heater (in-house-constructed). Bioreps 3–5 were analyzed with a Waters nanoAcuity UHPLC system (due to the failure of Easy-nLC) using the same gradient and flow rates and 60° C temperature; however, instrument configuration between the Waters UHPLC and Thermo MS instruments necessitated collecting MS data during the 30 min of sample loading, resulting in LC–MS raw data files of 150 min instead of 120 min for the Easy-nLC system. MS parameters were the same for all five bioreps. The nanospray source voltage was set to 2200 V. Full-mass profile scans were performed in the orbitrap between 375 and 1500 *m*/*z* at a resolution of 120,000, followed by MS/MS HCD scans in the orbitrap of the highest intensity parent ions in a 3 s cycle time at 30% relative collision energy at a resolution of 15,000 with a 2.5 *m*/*z* isolation window. Charge states 2–6 were included, and dynamic exclusion was enabled with a repeat count of one over a duration of 30 s and a 10 ppm exclusion width, both low and high. The AGC target was set to “standard,” maximum inject time was set to “auto,” and 1 microscan was collected for the MS/MS orbitrap HCD scans.

### MS Data Analysis

Mass spectral files were analyzed with the free and open-source search software program MetaMorpheus version 1.0.7 (https://github.com/smith-chem-wisc/MetaMorpheus).^59,60^ Databases included the reviewed Swiss-Prot mouse (canonical) database, a spectral library database for mouse proteins predicted by pDeep,^51,59,61,62^ and the MetaMorpheus default contaminants database; the combined search database contained 17466 nondecoy protein entries including 476 contaminant sequences. The following search settings were used: protease = trypsin; search for truncated proteins and proteolysis products = false; maximum missed cleavages = 2; minimum peptide length = 7; maximum peptide length = unspecified; initiator methionine behavior = variable; fixed modifications = carbamidomethyl on C and carbamidomethyl on U; variable modifications = oxidation on M; max mods per peptide = 2; max modification isoforms = 1024; precursor mass tolerance = ±5.0 PPM; product mass tolerance = ±20.0 PPM; report PSM ambiguity = true. A 1% FDR threshold was applied for both peptides and proteins.

To determine the protein interactome of the *Gas5* target lncRNA, protein abundances from the *n* = 5 captured samples were compared to the *n* = 5 scrambled controls using Perseus software.^63^ Briefly, the protein LFQ intensities from MetaMorpheus output were log 2-transformed, normalized by the median *Z*-score, and filtered to only include proteins having five valid values in at least one condition (effectively the “captured” condition for this data set). Missing values (effectively only the “scrambled” condition for this data set) were imputed from a normal distribution using a width of 0.3 and a downshift of 1.8 standard deviation units. A paired, two-sample Student’s *t* test was performed between the captured and scrambled conditions using an S0 value of 1. A permutation-based FDR calculation was performed, yielding 303 proteins having a *q*-value <0.01. These proteins were evaluated for Gene Ontology (GO) enrichment analysis using the publicly available GO analysis tool (http://www.geneontology.org/) and protein–protein interactor analysis using STRING.^64,65^ RBPmap (http://rbpmap.technion.ac.il/), which is a web server designed for predicting protein interactors of target RNA and mapping binding sites of RNA-binding proteins, was used for further analysis.^66^ The results from RBPmap were compared with those of our enrichment list of 303 proteins.

### RIP-Qpcr Analysis

The RIP-qPCR protocol was adapted from Martindale et al., with modifications outlined here.^67^ Cells are lysed in a buffer containing 20 mM Tris–HCl, pH 7.5, 100 mM LiCl, 5 mM MgCl_2_, 0.5% NP-40, 10 mM ribonucleoside vanadyl complex, 100 U/mL RNasin Plus, and 1× Halt Protease Inhibitors with a final cell concentration of 2.5 × 10^7^ cells/mL. Cells were lysed by frequent vortexing for 10 min, keeping the cells on ice between vortexes. The cell lysate was sonicated on ice for 24 s, with 4 s of rest between each 4 s sonication interval, using a full probe on a Fisher Scientific model 550 Sonic Dismembrator. The protein concentration was measured by bicinchoninic acid assay per manufacturer’s protocol. 300–500 μg of protein was used per experiment and brought to a volume of 200 μL. Samples were incubated for 10 min at 50 °C to denature proteins. Antibodies (2 μg) were added to each cell lysate and incubated overnight at 4 °C with rocking. Antibodies specific to each target protein (Cell Signaling Technology: Anti-YBX1 (9744S), Anti-hnRNPK (4675S), and Anti-eIF3c (2068); Abcam: Anti-hnRNPD (ab61193); Thermo Fisher Scientific: Anti-eIF3e (PA5–29487); Santa Cruz Biotech: Anti-Rps23 (sc-100837)) and negative control antibodies (normal mouse IgG, EMD Millipore, 12–371) were used. Protein A beads (25 μL; Life Technologies, 88845) were added to the lysate–antibody mixture and incubated for 1 h at 4 °C with rocking. Beads were collected using a magnet and washed twice with 500 μL of buffer. The remaining beads were resuspended in lysis buffer and treated with Proteinase K (10 μL; Promega, N2615), incubating for 4 h at 37 °C. RNA isolation and qPCR were performed as described.

## Results

### HyPR-MS Analysis of lncRNA–Protein Interactions

*Gas5* was reported to be DDX41-regulated in our previous study.^56^ *Gas5* has been studied in many different cancers but has not been extensively explored in the hematopoietic system, leaving its role in hematopoiesis unclear. Thus, *Gas5* was selected as a target for the lncRNA–protein interaction study. Among 149 transcripts of *Gas5* (https://useast.ensembl.org/Mus_musculus/Gene/Summary?g=ENSMUSG00000053332;r=1:160861992-160866116), *Gas5-224* was increased to 1.52-fold (*p* = 0.025) in HET-WT cells compared with HET cells and had sufficiently high transcript levels, measured in transcripts-per-million (TPM), consistent with the requirements for HyPR-MS analysis (Tables 2; S2 and S3). Thus, *Gas5-224* was utilized for the analysis of DDX41-regulated lncRNA–protein interactions. HyPR-MS was performed on WT cells to purify specific lncRNA–protein complexes and identify protein interactors.

**Table 2:** TPM Values of *Gas5*-224 in WT, HET, and HET-WT Cell Lines

*Gas5*-224	TPM			HET vs HET-WT	
	WT-empty (WT)	HET-empty (HET)	HET-WT	fold change	*P*-value
	268	232	352	1.52	0.025

After hybridization capture by COs and release by ROs, the released samples were analyzed by using RT-qPCR assays for RNA analysis or MS for proteomic analysis. Small-scale HyPR-MS experiments were performed using 10^6^ cells to assess the necessary parameters for large-scale implementation. According to the small-scale results, CO1 gave a higher average capture efficiency and higher average capture specificity than CO2 or the combination of CO­(1 + 2) (Supporting Information Note S2 and Figure S2). Note that one might expect the combination CO­(1 + 2) to always outperform an individual CO, but that does not have to be true for two reasons: (i) the combination condition employs only half as much of each CO, and (ii) there can be unexpected interactions between the COs, ROs, and target RNAs despite efforts to minimize these during the CO/RO design. Therefore, after this small-scale preliminary phase, large-scale HyPR-MS experiments were performed using CO1 alone. We conducted five biological replicates using 10^8^ cells and 500 pmol of CO1, as these levels showed the best performance in small-scale experiments (Table 3). RT-qPCR was utilized to quantify the amount of *Gas5* lncRNA in four types of samples: (1) initial lysate sample prior to the addition of COs (input); (2) lysate sample after the capture of target lncRNA (noncaptured); (3) sample isolated from the original lysate by biotin-beads using specific capture oligos (captured sample); and (4) sample isolated from the original lysate by biotin-beads using scrambled oligos (scrambled sample). Capture efficiency is defined as the percentage of the lncRNA target captured in the captured sample relative to the total lncRNA in the input lysate sample. Capture specificity is defined as the fold change of the captured lncRNA by specific capture oligos compared to the captured lncRNA by scrambled oligos. The capture efficiency of *Gas5* in the captured samples averaged 77% across all large-scale experiments. Additionally, the quantity of *Gas5* lncRNA in the CO1-captured samples was, on average, 40-fold (capture specificity) higher than that in the scrambled control samples (Figure 1). These results mirror those of the small-scale experiments, confirming that sufficient material was captured for robust analysis via MS.

**Table 3:** Small-Scale and Large-Scale HyPR-MS Experimental Design

	small-scale	large-scale
cells	1 × 10^6^	1 × 10^8^
bioreplicates	3	5
capture oligos (COs) used	CO1, CO2, CO1 + CO2	CO1
amount of COs	5 pmol	500 pmol
qPCR assays	3	3

**Figure 1:**
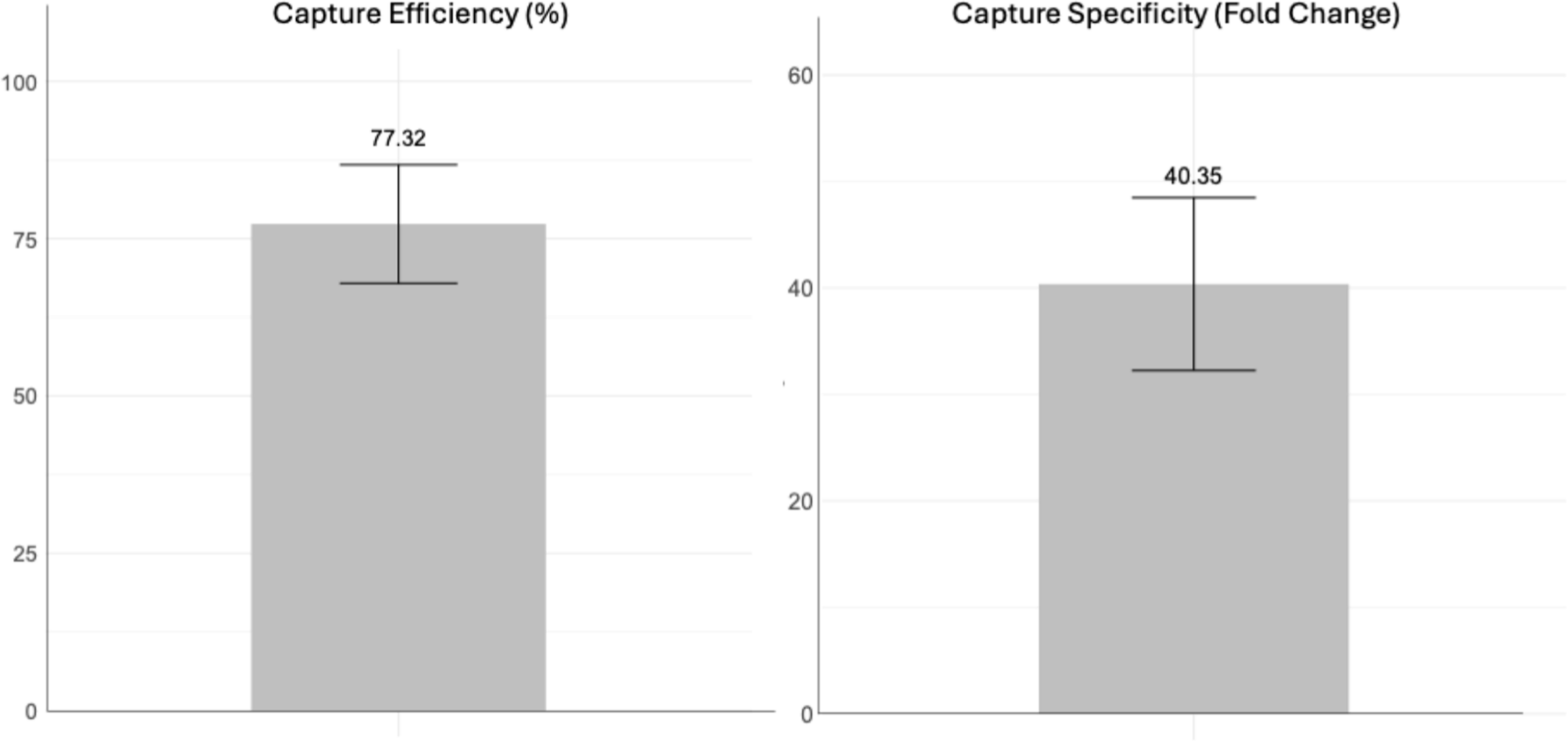
Large-scale HyPR-MS performance. Left panel: capture efficiency of CO1 was measured in large-scale HyPR-MS experiments. Right panel: capture specificity of CO1 in large-scale HyPR-MS experiments. Results and error bars were obtained from four biological replicates.

### Discovery of *Gas5* lncRNA Interactomes via MS Analysis

We conducted MS analysis to discover proteins that interact with *Gas5*. Five biological replicates were utilized, and for each replicate, we analyzed the *Gas5*-captured samples and the scrambled samples as a control, totaling ten samples. The raw data files were searched using MetaMorpheus. On average, 681 proteins were identified from the *Gas5*-captured samples, while an average of 272 proteins were identified from the scrambled control samples (Supporting Information Table S4). The MS proteomics data have been deposited to the ProteomeXchange Consortium via the PRIDE^68^ partner repository with the data set identifier PXD062695. Quantification analysis comparing the *Gas5* captured to the scrambled oligo control was then performed using Perseus (see details in the Experimental Section). Briefly, protein intensities were log 2-transformed, normalized, and imputation was performed for proteins quantified in the *Gas5* lncRNA captured samples but without quantifiable intensities in the scrambled oligonucleotide captured samples. A paired student’s *t* test was applied to compare protein intensities across all captured samples against those from the scrambled oligonucleotide controls. A permutation-based FDR calculation was performed, and proteins with *q*-value <0.01 were considered to be enriched in the captured samples. This analysis revealed the protein interactome associated with *Gas5* lncRNA, which included 303 unique proteins (Figure 2, Supporting Information Table S5).

**Figure 2:**
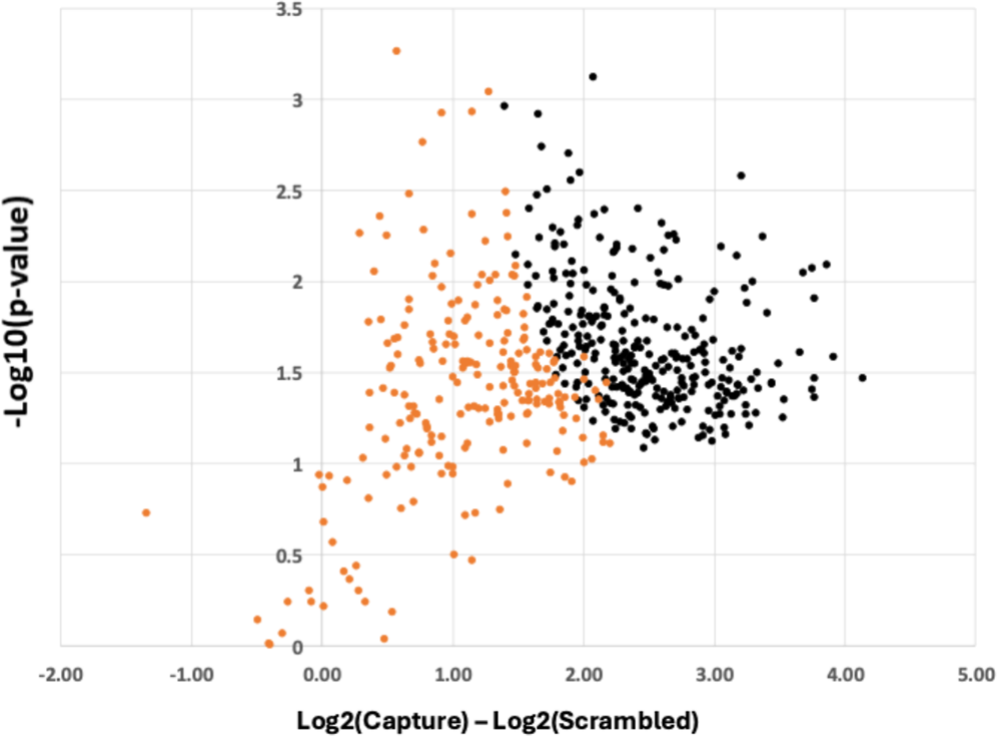
Quantitative proteomic results in a volcano plot. The 303 proteins significantly different between the captured samples and scrambled controls have black data points (*q*-value <0.01 by permutation-based FDR calculation). *X*-axis is approximately log 2 (fold change), calculated as the difference between the averages of log 2 (intensity). Data are provided in Supporting Information Table S5.

To understand the functions of these proteins, STRING was employed, and the functional classifications of some proteins are summarized in Table 4. Some of these proteins are consistent with the reported functions of *Gas5* or DDX41, which may provide insights into the mechanisms of how *Gas5* exerts its functions in the DDX41 regulated system. For example, hnRNPD and hnRNPK are implicated in cell cycle regulation, and therefore, these proteins may operate with *Gas5* to regulate the cell cycle. In addition, Ybx1 has a role in DNA/RNA processing, indicating that *Gas5* might interact with Ybx1 to regulate these processes.

**Table 4:** Predicted Functions of Some Example Proteins Identified by STRING

function	example proteins
protein synthesis and assembly	Eif3c, Eif3e, Rps23
cell cycle regulation	hnRNPD, hnRNPK, Psmd3, Mcm7, Hsp90aa1
apoptosis	Vdac1, Psmd3, Psmd14
DNA/RNA processing	hnRNPD, hnRNPK, Ybx1
translation regulation	Eif3c, Eif3e, hnRNPD, Ddx3x, Upf1
gene expression regulation and transcriptional control	Ybx1, Calr
maintenance of protein homeostasis	Psma6, Psmd14, Psma1, Psma2

### Evaluation and Validation of Proteins Identified by HyPR-MS

Our evaluation of these protein interactors encompassed three dimensions: comparisons with proteins suggested to bind *Gas5* in other biological systems, comparisons with predicted partners using a computational tool for identifying protein interactors of target RNAs, and enrichment analysis.

Several proteins have been reported to bind *Gas5* in other biological systems. Evaluation of whether these proteins were enriched in our analysis revealed two proteins, namely, Ybx1 (Y-box binding protein 1) and hnRNPK (heterogeneous nuclear ribonucleoprotein K).

We utilized RBPmap, a prediction tool for identifying the protein interactors of target RNAs and mapping binding sites of RNA-binding proteins, to predict the possible protein interactors for *Gas5*.^66^ The results from RBPmap were compared to the proteins that we detected. One of these proteins, hnRNPD (also known as AUF1), was predicted to be associated with *Gas5* by the RBPmap tool (Table 5).

**Table 5:** Predicted Binding Protein for *Gas5* Identified by RBPmap—hnRNPD/AUF1

protein	motif	position	*Z*-score	*P*-value
hnRNPD/AUF1	Uauuaa	289	2.561	5.22 × 10–3
		462	2.573	5.04 × 10^–3^
		467	2.976	1.46 × 10^–3^
		473	2.695	3.52 × 10^–3^
		475	3.012	1.30 × 10^–3^

GO enrichment analysis was performed, and the GO analysis considered three aspects of how gene functions can be described: biological processes, molecular functions, and cellular components. The interactome of lncRNA *Gas5*, as determined above, was evaluated using this tool for annotations statistically over-represented in the interactome relative to a random selection of the same number of proteins from the mouse proteome. The resulting over-represented annotations are listed in Table 6. Proteins interacting with *Gas5* are related to gene expression, cell cycle DNA replication, positive regulation of mRNA binding, and protein–RNA complex assembly, among other processes (FDR < 0.05).

**Table 6:** GO Terms Enriched with *Gas5* lncRNA Interactomes

category	GO term	protein #	FDR
biological process	gene expression	124	3.21 × 10^–31^
	“de novo” post-translational protein folding	11	2.56 × 10^–13^
	positive regulation of establishment of protein localization to telomere	8	3.68 × 10^–11^
	protein–RNA complex organization	20	1.36 × 10^–9^
	protein–RNA complex assembly	19	4.48 × 10^–9^
	cell cycle process	31	7.73 × 10^–4^
	positive regulation of telomerase RNA localization to Cajal body	3	9.02 × 10^–4^
	carboxylic acid metabolic process	47	9.64 × 10^–4^
	cell cycle DNA replication	5	2.01 × 10^–3^
	positive regulation of mRNA binding	2	2.48 × 10^–2^
	positive regulation of DNA metabolic process	21	3.33 × 10^–2^
molecular function	rRNA binding	22	7.05 × 10^–21^
	mRNA binding	37	2.02 × 10^–18^
	translation factor activity	14	8.55 × 10^–9^
	single-stranded DNA binding	13	2.82 × 10^–6^
cellular component	cytosol	200	1.06 × 10^–62^
	mitochondrion	100	1.36 × 10^–28^

STRING was used to visualize a network based on inferences from previously mapped proteins and their interconnections. Supporting Information Figure S3 shows a summary of interactions among the 303 proteins. Several biological processes were enriched, including regulation of translation, negative regulation of RNA splicing, and negative regulation of mRNA metabolic process (Figure 3A–C, respectively). These biological processes are closely related to the known functions of *Gas5* and DDX41, suggesting that our findings may uncover potential regulatory pathways through which DDX41 and *Gas5* exert their functions in the hematopoietic system. For example, DDX41 may regulate *Gas5* by interacting with hnRNPK to influence RNA splicing. Alternatively, DDX41 may modulate *Gas5*, which in turn regulates gene translation by interacting with proteins such as hnRNPD, Ybx1, and Eif3c.

**Figure 3:**
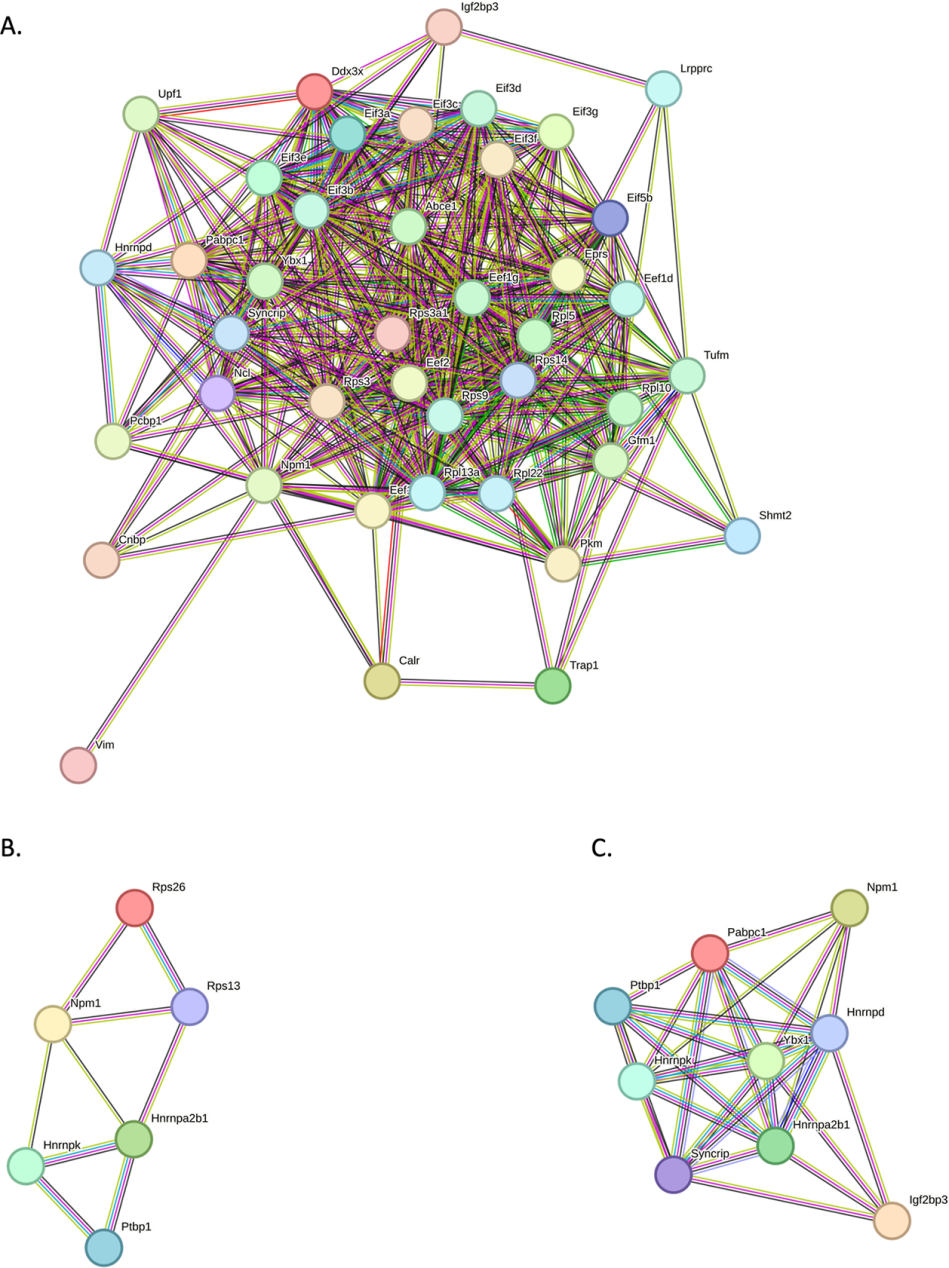
STRING analysis of the 303 proteins shows that these proteins are involved in known and predicted protein–protein interactions. (A) Interactions of proteins enriched in the biological process of regulation of translation. (B) Interactions of proteins enriched in the biological process of negative regulation of RNA splicing. (C) Interactions of proteins enriched in the biological process of negative regulation of the mRNA metabolic process. See Supporting Information Figure S3 for STRING interactions among all 303 proteins. Network nodes represent proteins. Lines of different colors represent seven types of evidence used in predicting associations. Red line: fusion evidence; green line: neighborhood evidence; blue line: co-occurrence evidence; purple line: experimental evidence; yellow line: text mining evidence; light blue line: database evidence; black line: coexpression evidence.

For further validation of *Gas5* protein interactors, we employed RIP-qPCR, a method complementary to HyPR-MS, that isolates target proteins and analyzes the associated RNA as a validation method. Six of the 303 identified proteins were selected for validation: Ybx1, hnRNPK, hnRNPD, Rps23, Eif3c, and Eif3e. Ybx1 and hnRNPK were selected because they have also been identified in other studies across various biological systems. hnRNPD was chosen based on its predicted interaction with *Gas5* identified by RBPmap, as well as its involvement in several enriched biological processes, including the regulation of translation. Rps23, Eif3c, and Eif3e were selected due to previous reports linking them to *Gas5*-dependent pathways or important biological processes such as the mTOR signaling pathway and mRNA splicing. STRING analysis revealed enrichment in several important biological processes, including negative regulation of mRNA metabolic process, negative regulation of RNA splicing, and regulation of translation, in which these proteins are involved either jointly or independently, supporting their selection (Table 7).^69–72^ The RIP-qPCR experiments compared the six targeted protein pull-down samples to IgG control samples. The results showed that *Gas5* was enriched (3–5-fold, *p*-value <0.05) in five of the six proteins (Figure 4). Thus, this orthogonal approach provided validation that *Gas5* interacts with Ybx1, hnRNPD, hnRNPK, Rps23, and Eif3e.

**Table 7:** Six Proteins Selected for RIP-qPCR Validation

protein	*q*-value	fold change
Ybx1	0	3.95
Rps23	0	3.89
hnRNPK	0	3.53
hnRNPD	0	3.09
Eif3c	0	3.05
Eif3e	0.00065	2.49

**Figure 4:**
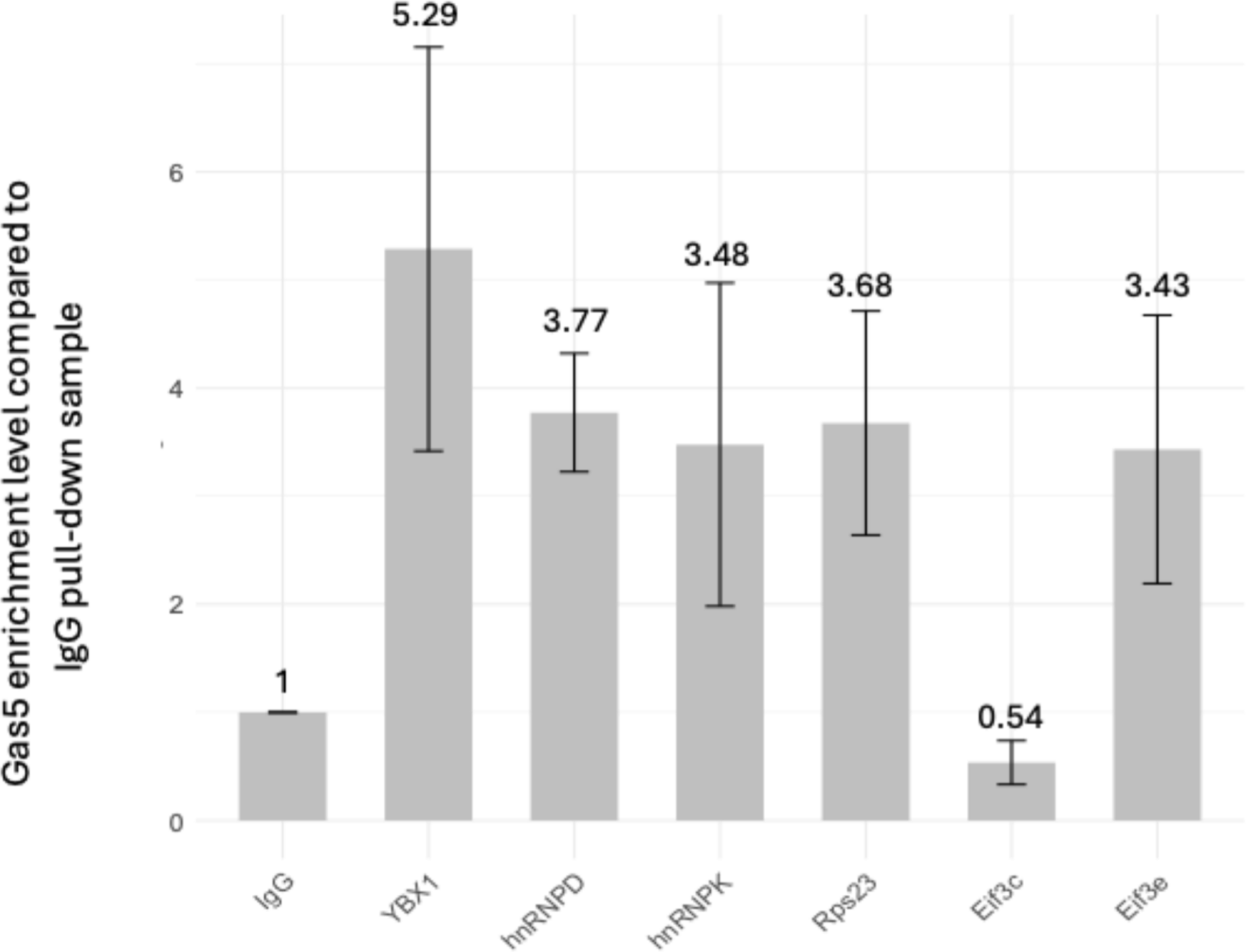
RIP-qPCR results validating five of six tested proteins. The figure shows the quantification of *Gas5* in the pull-down samples of six targeted proteins using RT-qPCR. The six proteins were pulled down from lysates, and the enrichment of *Gas5* was assessed relative to IgG control samples. The data represent the average fold enrichment of *Gas5* in the protein pull-down samples compared to the IgG control, with fold changes ranging from 3 to 5 and a statistical significance of *p*-value <0.05. Error bars indicate the standard deviation across three biological replicates.

## Summary and Discussion

Although DDX41 is a crucial regulator of hematopoiesis, little is known about the contribution of lncRNAs to its important activities. Previously, we demonstrated that DDX41 regulates *Gas5* levels.^56^ Among the multiple transcript isoforms of *Gas5*, *Gas5*-224 was selected as a target for the identification of *Gas5*–protein complexes, as it exhibited significant differences when DDX41 was expressed in the progenitor cell genetic complementation system. In addition, we predicted that *Gas5*-224 had a sufficiently high transcript level that would be compatible with HyPR-MS analysis.

Utilizing HyPR-MS, a technology known for its efficacy in discovering RNA-binding proteins, we purified lncRNA–protein complexes and employed MS to identify the interactors. Differential quantification analysis of proteomic data revealed 303 proteins, with their levels differing significantly between captured samples and scrambled controls. Among these, Ybx1 and hnRNPK had previously been identified as *Gas5*-interacting proteins in other systems. One study demonstrated that *Gas5* interacts with Ybx1, and *Gas5* knockdown increases Ybx1 protein turnover without affecting its transcription.^73^ This study suggested that *Gas5* downregulation reduced Ybx1 protein levels, which, in turn, decreased Ybx1-dependent p21 expression and abolished G1 phase cell cycle arrest in stomach cancer cell lines (HGC-27 and SGC-7901).^73^ In another study, overexpression of *Gas5* inhibited the PI3K/AKT/mTOR signaling pathway in ovarian cancer cells, and *Gas5* interacted with hnRNPK, affecting hnRNPK stability.^74^ This study also reported that *Gas5* inhibited ovarian cancer metastasis by downregulating hnRNPK expression.^74^

In addition to our RNA-binding protein-capture technology, we utilized RBPmap to predict the possible protein interactors for *Gas5*. The results from RBPmap were compared with those of the 303 proteins that we detected. hnRNPD was predicted to be associated with *Gas5* using the RBPmap tool. hnRNPD has been reported to be a regulator in adult muscle stem cells by targeted degradation of specific mRNAs such as ARE-mRNAs.^75^ Notably, hnRNPD (AUF1) is also a transcriptional target of p53, suggesting its involvement in p53-dependent mechanisms involving *Gas5*.^76^ Additional pathway and enrichment analysis via GO and STRING analysis indicated the enrichment of biological processes associated with mRNA splicing and regulation of translation. Furthermore, some of the proteins that we identified were found to be related to the *Gas5*-mediated signaling pathways, including the p53 network and the mTOR signaling pathway, according to other studies. Ybx1, hnRNPD, and hnRNPK are integral to the p53 network, each with distinct functions: Ybx1 was reported to interact with the tumor suppressor p53, inhibiting p53-dependent cell death and thus acting as a negative regulator of apoptosis;^69^ hnRNPD was found to be involved in destabilizing the mRNAs of p53;^77^ hnRNPK was reported to play a crucial role in the p53/TP53 response to DNA damage, functioning at both transcription activation and repression levels.^78^ Other identified proteins, such as Eif3c, Eif3e, and Rps23, were found to be involved in the mTOR signaling pathway, which regulates protein synthesis.^70–72^

Six proteins were selected for RIP-qPCR validation, including those identified in other systems, predicted by RBPmap, or related to *Gas5*-mediated signaling pathways. RIP-qPCR analysis confirmed five proteins, which are Ybx1, hnRNPD, hnRNPK, Rps23, and Eif3e, as *Gas5* lncRNA interactors compared with an IgG negative control. However, the RIP-qPCR results did not reveal the enrichment of Eif3c. Several factors may contribute to this lack of enrichment. First, the antibody used for RIP may have a low affinity or specificity for Eif3c, leading to inefficient immunoprecipitation. Second, Eif3c may be expressed at levels below those required for detection. Third, Eif3c may not stably interact with *Gas5* or may require specific conditions or cofactors that were not present under the conditions used for RIP analysis.

In summary, our studies revealed multiple protein interactors of DDX41-regulated LncRNA *Gas5*. Our findings suggest key hypotheses for future exploration: (1) DDX41, *Gas5*, and p53: DDX41 regulates *Gas5*, which in turn could function in the p53 network through its interactions with Ybx1, hnRNPD, and hnRNPK. This hypothesis posits that *Gas5* might regulate the cellular response to stress and DNA damage, potentially mediating the balance between cell survival and apoptosis. (2) *Gas5*, DDX41, and the mTOR pathway: DDX41 may also regulate *Gas5* to modulate the mTOR signaling pathway via its association with proteins, such as Eif3e and Rps23, to affect protein synthesis and cellular metabolism. In future studies, it will be instructive to test these hypotheses and compare DDX41-dependent *Gas5* regulation and *Gas5* function between physiological and pathological states.

## Supplementary Material





## Data Availability

The RNA-seq data set analyzed during the current study is available in the GEO repository (accession ID: GEO208400). The proteomic data set is available via the PRIDE repository in ProteomeXchange with identifier **PXD062695**.

## Notes

Sequences of COs, SO, and ROs; *Gas5*-224 sequence; top 20 abundant *Gas5* transcripts ranked by TPM values in WT cell lines; protein identification results of the *Gas5* captured and scrambled samples; materials and equipment; small-scale HyPR-MS experimental procedure; locations of capture oligos and qPCR assays at the *Gas5*-224 sequence; small-scale HyPR-MS performance; and summary of interactions among the 303 proteins analyzed by STRING (PDF)
Proteomic results of the 303 proteins showing significant differences between captured and scrambled samples (*q*-value <0.01) (XLSX)
