# Supplementary material for: Identifying lncRNA–Protein Interactions in Hematopoietic Progenitor Cells by Hybridization Capture and Mass Spectrometry
Source: J Proteome Res. 2025 Aug 1;24(9):4586–96. doi: 10.1021/acs.jproteome.5c00334 (PMC12345361; doi:10.1021/acs.jproteome.5c00334)
Supplement: Supplementary file 1 [file pr5c00334_si_001.pdf]

Supporting Information

Identifying lncRNA-protein interactions in hematopoietic progenitor cells by hybridization capture and mass spectrometry

Yuling Dai[1], Jeong-Ah Kim[2], Isabella T. Whitworth[1], Mark Scalf[1], Mabel M. Jung[2], Brian L. Frey[1], Emery H. Bresnick[2] & Lloyd M. Smith[1]*

[1]Department of Chemistry, University of Wisconsin-Madison, Madison, WI, 53706 USA

[2]Wisconsin Blood Cancer Research Institute, Carbone Cancer Center, Department of Cell and Regenerative Biology, University of Wisconsin School of Medicine and Public Health, Madison, WI, 53705 USA

Corresponding Author: Lloyd M. Smith smith@chem.wisc.edu

<u>Table of Contents</u>

Supplemental Table 1. Sequences of COs, SO, and ROs (toehold sequences are highlighted in red)

Supplemental Table 2. *Gas5*-224 sequence.

Supplemental Table 3. Top 20 Abundant *Gas5* Transcripts Ranked by TPM Values in WT Cell Lines.

Supplemental Table 4. Protein Identification Results of the *Gas5* Captured and Scrambled Samples (Five Biological Replicates)

Supplemental Table 5. Proteomic Results of the 303 Proteins showing Significant Differences between Captured and Scrambled Samples (q-value<0.01). Provided as an Excel spreadsheet.

Supplemental Note 1. Materials and Equipment

Supplemental Note 2. Small-scale HyPR-MS experimental procedure.

Supplemental Figure 1. The locations of capture oligos and qPCR assays at the *Gas5*-224 sequence.

Supplemental Figure 2. Small Scale HyPR-MS performance.

Supplemental Figure 3. Summary of interactions among the 303 proteins analyzed by STRING.

Supplemental Table 1. Sequences of COs, SO, and ROs (toehold sequences are highlighted in red)

	Length	Location	Sequence
CO1	25	160-184	CAATTGAC ATTTCATTTTCTGGTCTTCTATTCT
RO1	25	160-184	AGAATAGAAGACCAGAAAATGAAAT GTCAATTG
CO2	26	438-463	CAATTGAC TATATCTGACACCATCTTCTATTTGA
RO2	26	438-463	TCAAATAGAAGATGGTGTCAGATATA GTCAATTG
SO	29	N/A	TCGTATCT GTTCTTTATCTTTATGGTATCTCGGTATT
RO of SO	29	N/A	AATACACGAGATACCATAAAGATAAAGAAC AGATACGA

Supplemental Table 2. *Gas5*-224 sequence.

Transcript	Sequence
Gas5-224	AGCCTTTCGGAGCTGTGCGGCATTCTGAGCAGGAATGGCAGTGTGGACCTCTGTGATGGGACATCTTGTGGGATCT<br>CACAGCCAGTTCTGTGGCAAAGGAGGATGAAGGCTTACGAGGACTCGTCAGGAAGCTGGATAACAGAGCGAGCGC<br>AATGTGCTAGAATAGAAGACCAGAAAATGAAATGGTGGAGTTTGAGGCTGGATAGACAGTTTGAAAGTTAACTGGTTG<br>CATGCTTGTTCATTTGGCTGGCTTGCTTGGGTACAAATAATGGTTTGAATAAAGAAAGGTATTAATGGGTCACCTCAAG<br>TGAAGGCACTGCAAACACAATGATTGGTCATTCTGAATTTCCGGTCCTTCATTCTGAATTTCAAAGGCTCCTGTGACAA<br>GTGGACATGCAGTGACTGCACCTTTGTTTCTGAGGTGCCTGGATGGAGGCTCAAATAGAAGATGGTGTCAGATATATT<br>GTGTTAAAATTTTACCATTAAAGTGTATTATAACATGAAC

Supplemental Table 3. Top 20 Abundant *Gas5* Transcripts Ranked by TPM Values in WT Cell Lines.

Transcript_id	WT average	HET average	HET-WT average
ENSMUST00000162558.9_*Gas5*-224	267.94	231.74	352.32
ENSMUST00000159706.9_*Gas5*-210	59.80	59.53	86.09
ENSMUST00000240868.1_*Gas5*-228	44.48	34.77	60.89
ENSMUST00000244019.1_*Gas5*-244	32.71	17.43	36.41
ENSMUST00000247723.1_*Gas5*-342	28.80	14.55	18.39

ENSMUST00000247704.1_*Gas5*-331	27.77	8.84	22.06
ENSMUST00000161461.9_*Gas5*-220	18.09	7.70	14.10
ENSMUST00000247714.1_*Gas5*-336	17.79	34.73	37.08
ENSMUST00000247715.1_*Gas5*-337	14.62	14.16	18.03
ENSMUST00000161380.9_*Gas5*-219	13.72	7.17	10.84
ENSMUST00000247717.1_*Gas5*-339	11.38	5.36	6.81
ENSMUST00000247435.1_*Gas5*-280	10.66	6.49	1.42
ENSMUST00000242794.1_*Gas5*-238	9.78	6.16	13.41
ENSMUST00000242726.1_*Gas5*-237	9.65	11.40	17.66
ENSMUST00000065709.13_*Gas5*-201	9.28	5.19	4.40
ENSMUST00000242491.1_*Gas5*-236	7.59	7.01	15.11
ENSMUST00000247729.1_*Gas5*-345	5.27	1.84	6.62
ENSMUST00000247377.1_*Gas5*-276	5.19	3.56	3.34
ENSMUST00000246077.1_*Gas5*-254	4.85	0.62	2.23
ENSMUST00000247644.1_*Gas5*-310	4.41	3.21	8.52

Supplemental Table 4. Protein Identification Results of the *Gas5* Captured and Scrambled Samples (Five Biological Replicates)

Count of Identified Proteins	*Gas5* Captured Sample	Scrambled Sample
Biological Replicate 1	1289	705
Biological Replicate 2	675	274
Biological Replicate 3	559	155
Biological Replicate 4	358	105
Biological Replicate 5	524	119
Average	681	272

Supplemental Table 5. Proteomic Results of the 303 Proteins showing Significant Differences between Captured and Scrambled Samples (q-value<0.01).

Provided as an Excel Spreadsheet file.

Supplemental Note 1. Materials and Equipment

Reagents (all materials used prior to qPCR were RNase free)
Cells, 1% formaldehyde-treated for 10 minutes (small-scale: 1x 10^6 per condition; large-scale: 1x 10^8 per bioreplicate)
Ribonucleoside Vanadyl Complex (RVC) 200 mM

Dithiothreitol (DTT), solid
RNasin Plus Ribonuclease Inhibitor (Promega, Cat. No. N261B)
Halt™ Protease Inhibitor Cocktail (100X) (Thermo Fisher Scientific, Cat. No. 78430)
Lithium Chloride (LiCl) 8M
Tris-HCl pH 7.5, 4M
Lithium Dodecyl Sulfate, solid (LiDS)
Triton X-100 (10% w/v)
Nuclease Free Water
Capture oligonucleotides, biotinylated (designed through the IDT website tools)
Sera-Mag magnetic streptavidin microparticles (GE Healthcare, Cat. No. 30152105010150)
Release oligonucleotides (complementary to each capture oligonucleotide)
Tris-HCl pH 8.0, 10 mM
Ethylene diamine tetra-acetic acid (EDTA) pH 8.0, 0.1 mM
Proteinase K
TRI Reagent (ThermoFisher Scientific, Cat. No. AM9738)
Chloroform (100%)
GlycoBlue™ Coprecipitant (Invitrogen, Cat. No. AM9515)
Ethanol (100% and 75%)
Reverse Transcription Kit (Thermo Fisher Scientific, Cat. No. 4368814)
LightCycler 480 Probes Master qPCR Kit (Roche, Cat. No. 04707494001)
qPCR assays for several regions along each target (designed with IDT website tools)
Urea, solid
Deoxycholic Acid, solid (DCA)
HPLC water
Iodoacetamide, solid
Ammonium bicarbonate (200 mM and 50 mM)
Sequencing Grade Modified Trypsin, lyophilized (Promega, Cat. No. V5111)
Ethyl Acetate
Trifluoroacetic Acid (TFA)

Benchtop rocker
Benchtop vortex mixer
Mini-centrifuge
Model 550 Sonic Dismembrator (Fisher Scientific)
Benchtop centrifuge
-80H freezer
-20H freezer
Nanodrop UV-Vis Spectrophotometer
Incubator (37H)
Thermocycler
Roche LightCycler® 480 System
Magnetic separation racks for 2 mL tubes
Magnetic separation racks for 50 mL falcon tubes
Pipets
RNAse-free pipet tips

2 mL tubes, low-bind RNAse-free
50 mL conical falcon tubes, low-bind
50ml conical falcon tubes, low-bind RNAse-free
200 uL tubes RNAse-free
RNaseZap™ RNase Decontamination Wipes (Thermo Fisher Scientific, Cat. No. AM9786)
Parafilm
Bucket centrifuge
Centrifugal filter units (0.5mL, 50 kDa filters, Millipore Amicon-Ultra)
Savant Speed Vac Concentrator

Supplemental Note 2. Small-scale HyPR-MS experimental procedure.

We designed two capture oligos (COs) for *Gas5*-224 (Figure 1), which were assessed in a small-scale experiment focusing solely on RNA measurements. Three biological replicates were used. The capture efficiency of *Gas5* averaged 52.9% in samples captured using CO1, 41.1% in those captured with CO2, and 35.7% with the combination of CO1 and CO2 (Supplemental Figure 1A). The quantity of *Gas5* in the CO1, CO2, the combination of CO1 and CO2, captured samples were, on average, 32-fold,30.6-fold and 19.3-fold higher than in the scrambled control samples (Supplemental Figure 1B). Based on these results, CO1 demonstrated superior capture efficiency and specificity and was selected for subsequent large-scale HyPR-MS experiments.

Supplemental Figure 1.

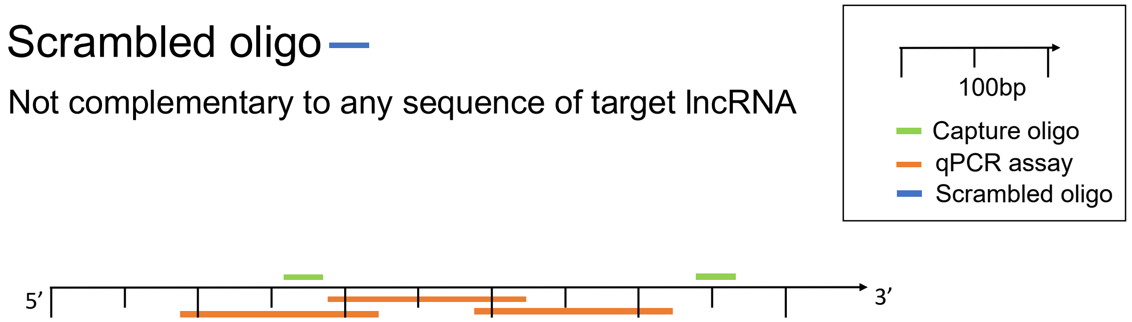

Supplemental Figure 1. The locations of capture oligos and qPCR assays at the *Gas5*-224 sequence. 2 captured oligos (green lines), 3 qPCR assays (orange lines), and scrambled oligo (in blue line) locations are shown.

Supplemental Figure 2.

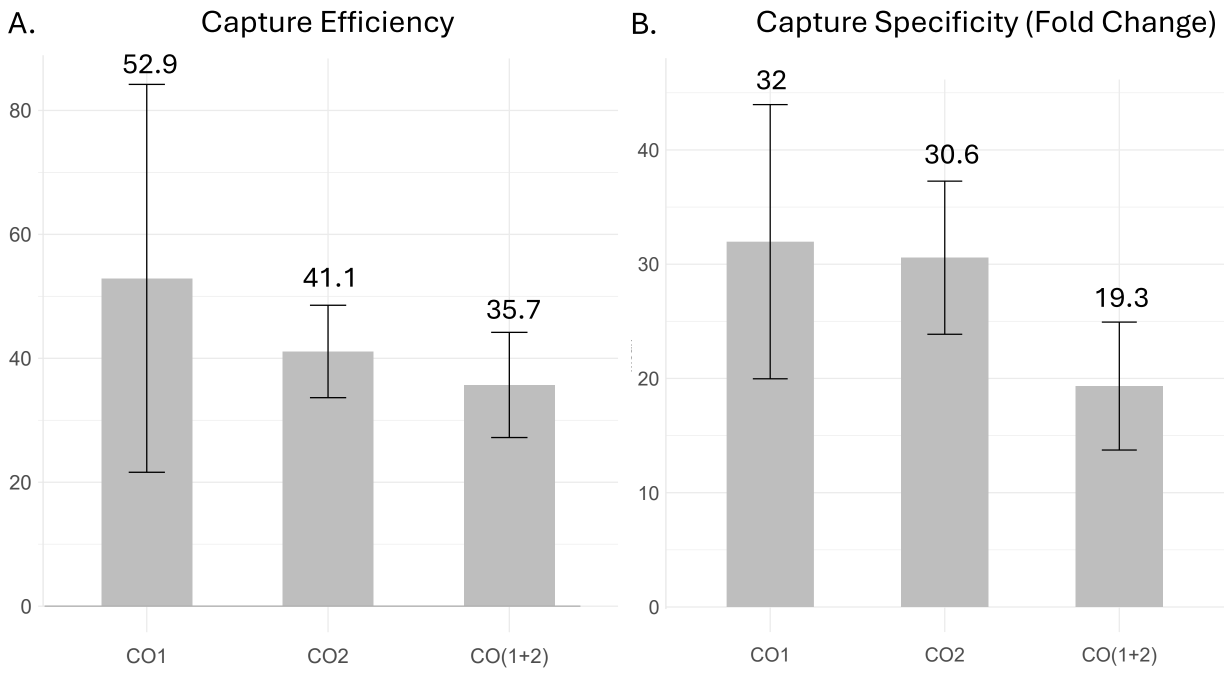

Supplemental Figure 2. Small Scale HyPR-MS performance.
A. Capture efficiency of COs in small scale HyPR-MS experiments.
B. Capture specificity of COs in large scale HyPR-MS experiments. In A and B, 3 biological replicates were utilized.

Supplemental Figure 3.

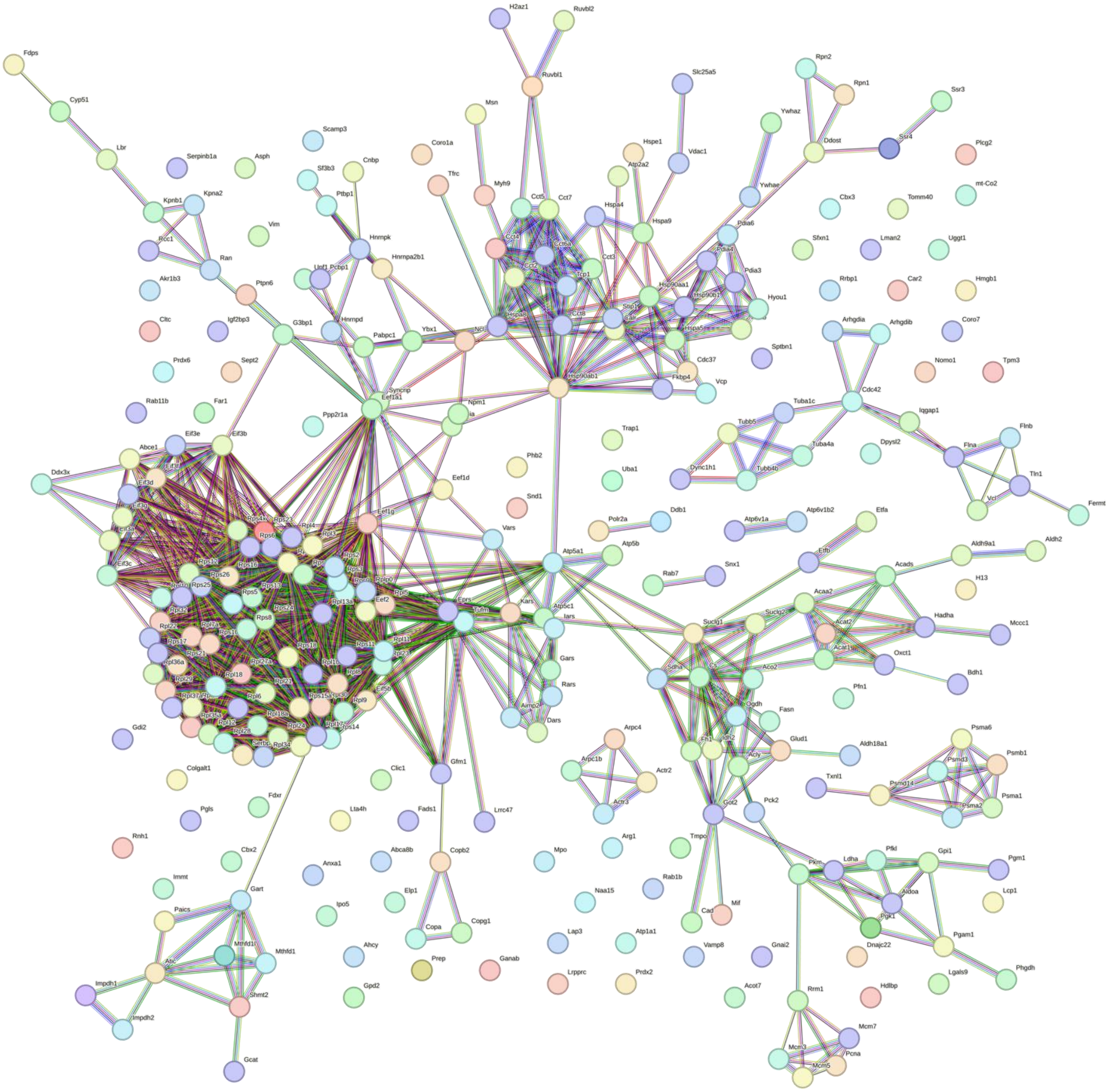

Supplemental Figure 3. Summary of interactions among the 303 proteins analyzed by STRING. Network nodes represent proteins. Lines of different colors represent seven types of evidence used in predicting associations. Red line: fusion evidence; green line: neighborhood evidence; blue line: co-occurrence evidence; purple line: experimental evidence; yellow line: text mining evidence; light blue line: database evidence; black line: co-expression evidence.
